# Unpacking the challenges of fragmentation in community-based maternal newborn and child health and health system in rural Ethiopia: A qualitative study

**DOI:** 10.1371/journal.pone.0291696

**Published:** 2023-09-21

**Authors:** Akalewold T. Gebremeskel, Ogochukwu Udenigwe, Josephine Etowa, Sanni Yaya

**Affiliations:** 1 School of International Development and Global Studies, University of Ottawa, Ottawa, Ontario, Canada; 2 School of Nursing, Faculty of Health Sciences University of Ottawa, Ottawa, Ontario, Canada; 3 The George Institute for Global Health, Imperial College London, London, United Kingdom; Sunu Sante Consulting, SENEGAL

## Abstract

**Introduction:**

In Ethiopia, country-wide community-based primary health programs have been in effect for about two decades. Despite the program’s significant contribution to advancing Maternal Newborn and Child Health (MNCH), Ethiopia’s maternal and child mortality is still one of the highest in the world. The aim of this manuscript is to critically examine the multifaceted fragmentation challenges of Ethiopia’s Community Health Workers (CHWs) program to deliver optimum MNCH and build a resilient community health system.

**Methods:**

We conducted a qualitative case study in West Shewa Zone, rural Ethiopia. A purposive sampling technique was used to recruit participants. Data sources were two focus group discussions with sixteen CHWs, twelve key informant interviews with multilevel public health policy actors, and a policy document review related to the CHW program to triangulate the findings. Thematic analysis of the qualitative data was conducted. The World Health Organization’s health systems framework and socio-ecological model guided the data collection, analysis, and interpretation.

**Results:**

The CHWs program has been an extended arm of Ethiopia’s primary health system and has contributed to improved health outcomes. However, the program has been facing unique systemic challenges that stem from the fragmentation of health finance; medical and equipment supply; working and living infrastructures; CHWs empowerment and motivation, monitoring, supervision, and information; coordination and governance; and community and stakeholder engagement. The ongoing COVID-19 and volatile political and security issues are exacerbating these fragmentation challenges.

**Conclusion:**

This study emphasized the gap between the macro (national) level policy and the challenge during implementation at the micro (district)level. Fragmentation is a blind spot for the community-based health system in rural Ethiopia. We argue that the fragmentation challenges of the community health program are exacerbating the fragility of the health system and fragmentation of MNCH health outcomes. This is a threat to sustain the MNCH outcome gains, the realization of national health goals, and the resilience of the primary health system in rural Ethiopia. We recommend that beyond the current business-as-usual approach, it is important to emphasize an evidence-based and systemic fragmentation monitoring and responsive approach and to better understand the complexity of the community-based health system fragmentation challenges to sustain and achieve better health outcomes. The challenges can be addressed through the adoption of transformative and innovative approaches including capitalizing on multi-stakeholder engagement and health in all policies in the framework of co-production.

## Introduction

Improving maternal, newborn, and child health (MNCH) outcomes has been an essential part of global health initiatives such as the Millennium Development Goals and the Sustainable Development Goals (SDGs) [1, 2]. There is a critical need to reinforce and optimize health systems in Sub-Saharan Africa (SSA). The reason for this is to respond to poor MNCH outcomes, achieve SDG #3, and ensure a resilient community health response [3] in the face of the ongoing COVID-19 pandemic and other public health emergencies [1, 2]) The SDG # 3 seeks to ensure “healthy lives and to promote the well-being of all at all ages” (UN,2015, p.14)) [2]. Specifically, major targets of this goal are devoted to MNCH: to reduce maternal mortality to less than 70 per 100,000 live births; to reduce newborn mortality to as low as 12 per 1,000 live births in every country; and to reduce Under-5 Mortality Rate (U5MR) to as low as 25 per 1,000 live births in every country [1, 2].

Despite significant progress in expanding community health services, gaps remain in improving access to MNCH for populations in Low- and Middle-Income Countries (LMICs), particularly in SSA countries including Ethiopia. The global Maternal Mortality Ratio(MMR) saw a reduction of 34 percent between 2000 to 2020 and U5MR reduction of 59 percent between 1990 and 2021 [4]. However, despite the progress, SSA countries continue to have the highest MMR and U5MR occurrences in the world and this has become a key health systems challenge in the region [5, 6].

Ethiopian U5MR stands at 67 deaths per 1,000 children and MMR stands at 412 per 100,000 live births [7]. To achieve the SDGs, Ethiopia must reduce neonatal deaths from 67 to 12 per 1,000 live births and maternal deaths from 412 to 70 per 100,000 live births in an environment of increasingly constrained health resources. This is one of the most daunting and urgent challenges facing the country as it seeks to ensure equitable MNCH and a resilient community health system. Worldwide, the pandemic is amplifying the problems of an already overwhelmingly fragile health system [7, 8]. According to WHO, COVID-19 could reverse decades of progress toward eliminating preventable maternal and child deaths [8]. Without a resilient health system, it will be impossible to achieve needed levels of MNCH outcomes during this era of the SDGs [7].

Ethiopia was one of the early adopters of the Alma-Ata Declaration of primary health care in 1978 [9]. The declaration emerged as a milestone towards addressing health system challenges and gross health inequalities between and within countries. The WHO identified community health workers (CHWs) as a key resource in providing basic health services for underserved areas because they help to fill the shortage of primary health service providers at the community level [9]. Since this time, CHWs programs have made significant contributions to enhancing access to basic primary health, especially for the disadvantaged, remote, and rural populations. CHWs’ provision of equitable MNCH services and corresponding outcomes are noticeable in SSA and internationally. For instance, rates of maternal, newborn, and child mortality and morbidity have dramatically reduced [10].

In the last two decades, Ethiopia has made various attempts to achieve universal primary health care and aspires to achieve Universal Health Coverage (UHC) by 2035 [6, 11]. Between 1995 and 2015, the country successfully completed the first 20-year health sector development program (HSDP). This program shifted the health system focus from predominantly curative to more preventive and promotive care, and it prioritized the needs of rural inhabitants, who make up 83% of the Ethiopian population [11]. In 2015, the government introduced its second 20-year strategy, the health sector transformation plan, focusing on primary health care and aligning with SDG #3 [12].

Ethiopia launched the CHWs’ Health Extension Program (HEP) in 2003 [12] as a key resource to enhance MNCH equity and provide basic health services for people in underserved rural areas where most of the population lives. The CHWs/ HEP is a nationwide community-based health program that integrates health workers into the national health system through two to three years of college-level training. CHWs are full-time employees. In agrarian areas of the country two salaried HEWs, who are women, cater to an average of 3000 to 5000 people per village (*kebele*) and have designated equipped Health Post (HP). The program was originally designed to provide over 16 packages of essential health promotion, disease prevention, and selected curative services [13]. Most of the CHWs’ role is associated with the provision of MNCH services including screening pregnant women, Antenatal Care (ANC), referring them to health centres for delivery, Postnatal Care (PNC), breastfeeding, vaccination/ immunization, family planning, hygiene and sanitation [14]. In Ethiopia, CHWs are also known as health extension workers (HEWs). HEWs are the frontline health workers for Ethiopia’s primary health care system [12–14]. We will use HEWs and CHWs interchangeably in this manuscript.

Over the course of two decades, multiple efforts have been made to enhance the responsiveness of the CHWs program including expansion to pastoralist and urban settings, improving community participation, and expansion to provide treatment of uncomplicated conditions [12]. In 2020 the Federal Ministry of Health (MoH) launched a roadmap for optimizing the HEP through redefining health service packages, task shifting, and changing the professional mix to join LMICs in the goal to achieve UHC by 2025. The program has grown to encompass 18 essential health service packages with a workforce of 40,000 HEWs working from more than 17,000 HP [12–14]. Throughout history, although often under a different name, HEWs/CHWs have been instrumental in providing communities with education on a myriad of issues. They also serve as key connections to various social services and healthcare resources [14]. A fundamental characteristic exuded by CHWs is their capacity to bring cultural relevance and sensitivity to each interaction. Because CHWs tend to come from the same communities they serve, they play a key role in insular settings where communities may experience fear and lack of trust. CHWs have the unique ability to distill the complexities for community members as they attempt to enter the healthcare system [6, 11, 14–16].

The Ethiopian CHWs/HEP program is the component of the primary health system at the community level that reaches rural communities by recruiting and training paid CHWs [12]. The primary healthcare units are designed to serve approximately 25,000 people. The country has a top-down decentralized model for the delivery of healthcare along political structures, with shared government responsibilities between the Federal MoH, the regional health bureaus, zonal health offices, and woreda(district) health offices. The health sector is structured into a three-tier system: tertiary care, provided at specialized hospitals; secondary care, provided at general hospitals with inpatient and ambulatory services; and primary healthcare (primary hospitals, health centers, rural regions, CHWs) [12–14].

Ethiopia has achieved remarkable health outcomes in the last two decades and CHWs/ HEWs are one of the major contributors to the success. The achievements include a 5% increase (from 39% in 2016 to 44% in 2019) in the percentage of children aged 12–23 months who received all basic vaccinations; the reduction of neonatal mortality rates from 49 per 1,000 live births in 2000 to 30 per 1,000 live births in 2019; a decline in U5MR from 123 deaths per 1,000 live births in 2005 to 59 deaths per 1,000 live births in 2019. Receipt of ANC and Institutional delivery has gone up from 27% and 5% in 2005 to 74% and 48% in 2019 respectively. The proportion of women with the recommended four or more ANC visits increased from 12% in 2005 to 43% in 2019 [17].

However, based on the Demographic and Health Survey in 2019 [17], there is a fragmentation in MNCH outcomes. Despite the progress that has been made, Ethiopia has a large burden of communicable diseases and still has a MMR of 412 per 100,000 live births and U5MR of 67 per 1,000 [7]. For example, while a higher proportion (74%) of mothers received the first ANC, only 43% received the recommended four or more ANC. Also, Skilled Birth Attendance (SBA) during childbirth is 48% PNC is at 34% of women aged 15–49 [17].

We argue that the CHWs program is organized to address immediate short-term outcomes including MNCH outcomes while ignoring the need to build a sustainable and resilient health system. Evidence suggests that the Ethiopian health system is in a fragile state [18, 19]. The system is not providing sustainable quality service, nor has it evolved to meet increased demands. High rates of inequities persist within the country in terms of health access and health outcomes. In Ethiopia, like in many other LMICs, the rural community health program has a complex political economy, consequently, huge disparities in the availability and utilization of MNCH services, and MNCH outcomes exist between rural and urban residents [18, 19]. Inequality in MNCH outcomes is increasing over time. The demand side factors that drive inequality in MNCH outcomes in Ethiopia include low economic status, illiteracy, rural residence, no occupation, and lack of access to mass media [18, 19]. The most disadvantaged women are the poor, rural residents, uneducated, unemployed, and those with low access to information [18, 19]. The health program has overwhelmingly failed to address the quantity and quality of health workers [18, 20]. The CHWs program continues to experience multiple challenges to enhance health outcomes [10, 13, 15, 16, 21–23].

In several countries of the world, health system fragmentation is becoming a common challenge, but its magnitude and primary causes may differ. Fragmentation could be described in different ways from different aspects of the health sector and beyond. For example, from a healthcare perspective, fragmentation means having multiple decision-makers make a set of healthcare decisions that would be made better through unified decision-making [24]. In the context of the CHWs program, ‘fragmentation’ is the interruption, glitches, and aborts of means of delivery of optimum MNCH and foundations of CHWs. Fragmentation manifests itself as a lack of coordination and consistency among the different levels, settings, organizations, functions, coordinated financial and resource management/governance systems, comprehensive engagement approach, and system thinking to ensure reliable and sustainable service and outcomes [25–28]. It may occur in settings where public health decisions are made based on limited or lack of evidence [29].

Many health systems in LMICs remain fragmented [29], however, primary health program fragmentation is less acknowledged among researchers and policy actors. Fragmentation has been discussed in global health literature, agendas, between prominent global health actors, and even in strong economies but often from a high-level perspective [21, 26, 29, 30]. Fragmentation is an underlying problem, it is a barrier to advancing UHC and a challenge to achieving SDGs [31–33]. Fragmentation by itself or in conjunction with other factors can lead to complications in access to services or delivery of services, inefficient use of resources and increases in production costs [26].

The fragmentation of essential pillars of the CHWs program is disruptive and problematic. Although efforts and debates continue regarding how to achieve reduction in adverse MNCH outcomes in line with the SDGs, there is limited evidence on what drives or causes fragmentation in health system. While the challenges of the CHWs program are multilevel and multidimensional, the existing evidence focuses on the knowledge, performance/role and effectiveness of CHWs. There are also quantitative and qualitative studies based on particular projects or sites, however, they do not consider the multilevel and multidimensional context of the CHWs program [15, 16, 18, 19, 34–37]. Comprehensive evidence on the multilevel and multifaceted challenges affecting the CHWs program’s effectiveness is limited. Furthermore, there is limited evidence on the ways in which the CHWs program’s fragmentation challenges have harmed and continue to harm MNCH, and the resilience of the community health system. The aim of this manuscript is to critically examine the multifaceted fragmentation challenges of Ethiopia’s CHWs program to deliver optimum MNCH and build a resilient community health system.

## Theoretical framework

Our study was guided by different theoretical frameworks which informed the research processes and research questions. First, we used the socio-ecological model (SEM) to inform the description of the multilevel determinants of the CHWs program’s effectiveness [16, 38]. The socio-ecological framework posits that factors at various levels uniquely and jointly contribute to health interventions. The levels are: (i) Health workers’ individual-level which includes their knowledge, skills and attitudes; (ii) Interpersonal factors, such as health personnel collaboration and supportive approaches; (iii) Community-level factors/informal institutions, such as community acceptability/recognition, trust, and community tradition, value and beliefs; (iv) Health system and logistics related factors, such as health policy, program, human resource/training, financial and material supply chain and logistic approaches [16, 39, 40].

Second, our study was informed by the WHO’s Health System Framework, a leading structure of discourse on health systems building. The framework comprises six operational building blocks—service delivery, health workforce, information, medical products and technologies, financing, and leadership and governance, it however falls short in including macrolevel community context and engagement. Despite this limitation, the framework’s relevance is recognised by policymakers, programmers and researchers in national and global health and has become a pillar for planning, implementation, and evaluation [16].

Our findings were reported based on the Consolidated Criteria for Reporting Qualitative Research (COREQ) (see S1 File).

## Methods

### Study design

This study is a qualitative case study using document reviews, Focus Group Discussions (FGDs)) and Key Informant Interviews (KII). Qualitative case study is a research methodology that helps in the exploration of a phenomenon within some context. Desai and Potter [41] stressed that development study requires the use of a wide range of research methods: the mix of methods enables the different techniques and their results to be compared against each other, allowing judgements to be made as to which method (or combination of methods) is the most appropriate for any particular purpose.

Yin [42] proposes that a case study inquiry should rely on multiple sources of evidence, because data source triangulation allows the researchers to have a trustworthy groundwork for the findings and the contribution of knowledge [42]. According to George and Bennett [43], case studies enables deep contextual understanding and they have potential for achieving high conceptual validity. Case studies are a useful means to closely examine the hypothesized role of causal mechanisms in the context of individual cases. The move from single-case to paired comparison offers a balanced combination of descriptive depth and analytical challenge that progressively declines as more cases are added [44]. In a case study, a real time phenomenon is explored within its naturally occurring context, with the aim of answering the “how” and “why” questions [42, 45].

The case focused on MNCH programs led by CHWs/ HEWs in rural Ethiopia. The choice to pursue this research as a case study is an attempt to better understand and explain how to capitalize and scale up on CHWs/ HEWs-led MNCH program to optimize health outcome and a resilient health system in rural Ethiopia. The study site was selected because of the opportunity it offers to study the CHWs program within its naturally occurring context. Furthermore, the lead author (AG) has extensive knowledge and work experience in the area and program to better understand and explain the case [46].

### Research setting

Ethiopia is one of the most populous countries in Africa with 109 million inhabitants, which is second to Nigeria. The World Bank [47] classifies Ethiopia as a low-income country, but it is also one of the fastest growing economies in SSA. It still has a largely rural population, above 80% of the population lives in rural areas, and 12–14% of the total population are pastoralists or agro-pastoralists [48]. Since the adoption of the 1994 ethnic based constitution, the government of Ethiopia has been structured in the form of ethnic- based federal system of government. The country is administratively divided into eleven ethnic-based regional states (provinces) and two chartered cities that are administered separately from states [49]. This study was conducted in Oromia regional state, which is the largest of the eleven Ethiopian states, both in terms of population and landmass [49].

Ethiopia has a top-down decentralized model for the delivery of healthcare along political structures. The health sector is structured into a three-tier system: tertiary care, provided at specialized hospitals; secondary care, provided at general hospitals with inpatient and ambulatory services; and primary healthcare (primary hospitals, health centers, and HEWs post) [50]. Each primary healthcare unit serves approximately 25,000 people. The CHWs/HEW program is the primary health system component at the community level that reaches the rural majority communities by recruiting and training paid community based HEWs [12].

In this research, the nested nature of the case study encompasses two distinct populations including HEWs/CHWs (16 FGD participants) on-site service providers, and subnational and national public health policy actors (12 KII participants). These populations are spread over three geographical areas with respect to the scope of their work: West Shewa Zone CHWs, Oromia Region (sub-national) and Ethiopia (national) public health policy actors.

The FGD data was collected from HEWs in West Shewa Zone. West Shewa is one of the zones of Oromiya region, the zone has 23 districts and is located to the west of Addis Ababa (Capital city of Ethiopia). The zone has a human population of 2,058,676, of which 1,028,501 are males and 1,030,175 are females. In this Zone, in 2019, 84 percent of the population lived in rural areas [51]. Currently, the zone has 8 hospitals, 92 health centers and 528 CHW posts (each post has an average of 2 HEWs). The CHW program and health posts are administered and funded under the public health system. Despite the progress in other parts of the country, the West Shewa zone is still underperforming when it comes to MCH outcomes.

### Characteristics of participants

#### FGD participant (Inclusion criteria)

All participants, CHWs/HEWs had to be adults ranging from the ages of 21 and who had been residents of the district (work site). The participants must have had one-year experience working as a CHW in MNCH before COVID-19; three or more years of CHW/MNCH work experience in rural areas; over one year of HEWs national training, must be a full time employee and be salaried. The study did not exclude HEWs/CHWs by their gender or sex.

#### Key informants

Key informants were recruited based on their known involvement in the policy process leading to the planning and implementation of the CHWs/ MNCH program. Snowball sampling was used to recruit participants whereby participants were suggested and recruited by other participants [52]. The principal investigator (AG) estimated the saturation of data between 10–12 interviews and 2 focus groups and 6–8 individuals based on previous similar approaches [53].

### Participant recruitment

#### Focus group participants

Using purposive sampling, AG worked with the Oromia Regional Health Bureau (ORHB) and zone health administration to select the two districts. Then AG contacted and worked with the district health office in an attempt to recruit potential FGD participants to send the recruitment poster to potential participants via the districts’ regular means of communication, telegram, email, and announcements during review meetings.

#### Key informants

The principal investigator, AG, worked with the MoH, ORHB, zone health administration to select the first potential participant in the KII.AG started by sending an invitation email with information about the study and the KII recruitment poster. The source of the referral (the referee) was confidential. The potential invited participant had the right to accept or decline the invitation, and this response was confidential too.

We used a first-come-first-served basis for enrolment. Prior to participating in this study, participants were asked to provide their free and informed consent by signing a consent form.

### Data collection

The data collection was organized into three parts: FGDs, IDIs, and document reviews. AG collected the data using semi-structured FGD guides developed in English and translated to the Oromo language and KII guides developed in English and translated to Amharic and Oromo. The guides were carefully crafted following the theoretical frameworks and relevant literature to include neutral, non-biased, and non-leading questions to avoid influencing participants’ responses. AG is fluent in the local language (Amharic and Oromo). Based on their agreement, AG contacted participants for their convenient time and place to sign the consent form and then conducted the FGD and KII. AG acted as the facilitator to recruit participants, ensure confidentiality, and distribute and collect consent forms.

At the beginning of each FGD and KII, AG briefly described the purpose of the study to the participants. All FGDs and 11 KII were conducted face-to-face and 1 KII was conducted using Zoom. After consent was given, each FGD and KII was audio recorded. The focus group sessions lasted between 80 to 90 minutes and the KII lasted between 60 to 90 minutes. A research assistant was assigned to take notes during the FGDs after a brief training on research ethics and processes provided by AG. The research assistant was fluent in the local Oromo language and possesses a BSc degree and research-related experience.

As a token of appreciation, all FGD and eight KII participants were compensated with a small honorarium, for their time and effort and to cover their lunch-related expenses. In the context of COVID-19, participants adhered to public health guidelines including wearing masks.

Most of the data collection was conducted face-to-face in Afan Oromo and Amharic, based on participants’ language preferences. Two FGDs were conducted with CHWs working in two different districts/ *Weredas* (*Adea Berga and Ejere*) of West Shewa Zone. In-depth semi-structured KIIs were conducted with twelve key informants from national and subnational level health systems. Data collection period was between mid-September 2022 to end of November 2022.

Data collection took place at different locations depending on the participants. The FGDs were conducted in a rented hotel hall while KIIs with policymakers were conducted in convenient locations for participants such as their offices.

We conducted a document review to enrich the findings from the FGD and KII, and to understand the context and operation of the HEWs/CHWs program. Policy documents, including national/regional strategies and plans, were considered to represent the major priorities for CHWs-led MNCH. The relevant and available documents were selected and accessed through the recommendation of the key informants working in the National and subnational HEP/MNCH program in Ethiopia.

### Data analysis

The data analysis process began with the development of a coding framework informed by the KII and FGD guides, the WHO health system framework and SEM, and the review of the existing literature. We followed Braun and Clarke’s [54] six step thematic analysis that includes: (1) familiarizing ourselves with the data; (2) generating initial codes; (3) developing a coding tree to guide the coding of transcripts; (4) identifying themes; (5) reviewing, defining and naming themes; (6) interpreting the narratives and stories and producing the report–a concise, coherent, logical, and non-repetitive account supported by vivid examples. FGDs and the KIIs were audio-recorded and AG (the principal investigator, PI) transcribed them verbatim. Verbatim transcription was done by listening to audio-recorded material. The two FGDs and four KIIs were in the Oromo language, and eight KII in the Amaharic language. They were transcribed into Word documents. Then, the transcribed documents in both languages were translated to the English language. Data were also cross-checked by listening to the original recording while reading along in English.

The transcript content was coded to ensure quality and accuracy, and reduce any discrepancies while coding transcripts. Codes were developed after an initial review of the transcripts. The transcript content was analyzed inductively to get familiar with the data. First, after familiarization with the transcript, AG & OU coded one transcript from each FGD and KII, they coded individually and compared their codes. After debriefing and consensus, the transcript was coded accordingly. After debriefing, JE and SY randomly chose and compared to check alignment or discrepancies of the coded transcript. Emerged themes were based on an iterative process of inductive and deductive approaches. Deductive coding approaches drew from existing literature including the systematic review on the same topic, and the study’s theoretical frameworks. In inductive approaches, themes emerged from the data and were not forced to fit into preconceived categories [55]. The analyses used triangulated data from multiple sources including multilevel government and non-government health policy experts, frontline CHWs, and document analysis. Given the small size of the data, we decided to code manually without employing any qualitative data analysis software.

### Policy documents

Policy documents were significant to the study and were used mainly from a realist perspective [56], that is, as a means to understanding the CHWs/HEP policy/ program in the context of rural Ethiopia. Hence, policy documents were essential in providing background information to the study and in defining the questions and trajectories that were pursued in the FGD and KII. The analysis was also enriched by useful insights from the policy document. Using thematic analysis, we sought documents that mention the major themes that emerged from the KII and FGD, based on inductive and deductive approaches. Major Health policy documents used in this study include the second Health Sector Transformation Plan (HSTP-II) [11], A roadmap for optimizing the Ethiopian HEP(2020–2035) [50] and more, see the list of policy documents (see S2 File).

### Trustworthiness

Trustworthiness, qualitative validation criteria were applied in this study in line with established guidelines [42]. Therefore, the researcher’s inherent bias was controlled partly by being self-aware and by allowing the participants to generate data based on their own perceptions. To enhance the trustworthiness of the study’s findings, we used different techniques recommended by renowned qualitative researchers including member checks and peer review [57], triangulating the data by using multiple sources of data to confirm emerging codes and themes [57], to serve as a data validation strategy and help to establish the credibility of the study findings [58].

### Ethical considerations

The research proposal was carefully evaluated, and the ethics of the study were subsequently reviewed and approved by two respective institutions, the University of Ottawa Research Ethics Board, ethics clearance certificate (Ethics File Number S-06-22-8072) and the Ethiopian Public Health Institute, Institutional Review Board (EPHI-RIB) certificate of approval (protocol number: EPHI-IRB-462-2022, minute No:109). In addition, to strengthen the importance of the study, support letters were received from the School of International Development and Global Studies, University of Ottawa, and form the study area, Ethiopian MoH, ORHB and West Shewa health office.

Participants gave their free and informed consent to be enrolled in the study. Participants provided written informed consent prior to participating in this study. They were also informed that once they chose to participate, they could withdraw at any time or chose not to answer any questions, to which there would be no negative consequences.

### Reflexivity and positionality

To enable any audience of qualitative studies to evaluate the validity of conclusions extrapolated from data, researchers should, as part of the study, neutralize or bracket their biases by stating them explicitly to the full extent possible [57]. AG, OU, JE and SY have extensive experience as global health experts and have multiple publications and work on MNCH and related policy and programs in SSA including Ethiopia. AG has more than eight years in community health program management in Ethiopia and worked in different contexts of health program implementation and evaluation in Ethiopia.

## Results

### Characteristics of participants

The sociodemographic characteristics of FGD participants is summarized as follows: In the FGDs, there were a total of 16 Female health extension workers from two districts (Adea Berga and Ejere) of West Shewa Zone. All the FGD participants are female and the majority 15(93.75%) of them are in the age range of 25–40. The highest level of education or training that they have completed were as follows: college diploma 12(75%) and over one year of HEWs training 4(25%). Participants’ total work experience as a HEW (in years): six and above 14(87.5%) and four to five years 2(12.5%). For all the participants, walking was their most used mode of transportation during field work/ outreach activities. The average distance between their work site and fieldwork/outreach site in kilometers: above 5km, 9(56.25); 3km to 5 km 5(31.25%); and less than 3 km 2(12.5%). The most used form of communication was face-to-face with clients during home visits for all the participants. In the past 12 months, 9(56.25%) of the CHW participants had an estimated monthly personal income of 5001 and above in Ethiopian Birr (ETB) and the rest earned an income below 5000 ETB.

A total of twelve public health policy/ program experts were recruited to participate in the KIIs. Eight participants (66.6%) were recruited from the three levels of government health structures. These include three participants who were recruited from MoH, they act as national primary health system and HEP experts; two participants who were recruited from ORHB, they act as regional primary health and HEP experts; and three participants who were recruited from West Shewa Zone (one Zonal level health MNCH expert) and two district level health office HEP experts. Four (33.3%) participants were recruited from NGOs. These include two National level experts (one program director and one M&E expert) and two from the regional-level program manager/ expert. In terms of gender, four (33.3%) women and eight men (66.6%) participated. Most of the participants have post-graduate level (MSc) educational backgrounds in health and related study programs and they have extensive (more than 10 years) public health-related experiences in different levels of responsibilities.

Using multilevel data and thematic approaches, our analysis uncovered underlying challenges for adequate MNCH service and outcome and fragmentation of community health program pillars (enabling situations). Participants indicated that despite the widespread belief that they are an extended arm of the health care system, multiple challenges in the CHWs program structure diminish their roles. We identified seven distinct yet interconnected sub-themes(sets) of fragmentation challenges in the community health program pillars and system. The results suggest that all the multilevel participants perceived multidimensional challenges of fragmentation in the CHWs program. These fragmentation challenges hindered the delivery of equitable, sustainable, and quality MNCH in rural Ethiopia. Fragmentation of community health program pillars includes fragmented health finance, fragmented medical and equipment supply, fragmented working and living infrastructures, fragmented CHWs empowerment and motivation, fragmented monitoring, supervision and information, fragmented coordination and governance, and fragmented community and stakeholder engagement. The ongoing COVID-19 pandemic and volatile political and security challenges in the country are exposing and exacerbating the fragmentation challenges. We present the seven distinct yet interconnected sub-themes(sets) of fragmentation challenges in community health program pillars as follows and for more perspective, See more quotes on S3 File.

### Fragmented health finance

Fragmented and inadequate health finance and resource allocation are the most critical challenges facing the CHWs program optimization. Health financing includes how all money/ resources are mobilized and used to improve the health and well-being of individuals. Health financing systems can affect access to healthcare in terms of quantity and quality. Government and non-government actors are responsible for the larger share in financing the CHWs program in Ethiopia.

However, participants revealed a lack of prioritization of health financing and a budget shortage from the government treasury. They also described fragmented funding from donors for MNCH and other community-based planned activities.

“*The overall health sector budget is limited; If we look at health finance*, *it is not better than other sectors; there is inadequate funding or budget allocated for CHW program*.*” (KII 2*, *National level NGO*, *Program Director)*

“*…But there is a budget shortage and capacity limitation*, *it is very difficult to accomplish what you have planned*, *including maternal and child health*. *It requires a big budget*.*”* ***(****KII 5*, *Regional health bureau*, *Primary health expert)*

Participants explained that the Ethiopian health system is dependent on donor support. Despite the higher demand for funding due to prevailing problems including high maternal and child morbidity and mortality, the available funding are fragmentated, small, declining and project-specific. Also, there is a lack of transparency and duplication in the management of available funds.

“*Funds have declined significantly*. *The health extension program is particularly affected*. *That is where most of the problems come from*. *The health extension program is at the primary scale because they think it is a successful program*. *This funding is said to have decreased*. *I think the reason for the decline is related to the existing political crisis in the country (the war in the northern part of the country)*. *It is not only impacting the health sector*, *but other sectors have been affected*.*” (KII-1*, *MoH*, *National level primary health program expert)*

“*Okay*, *compared to other sector*, *there is a large number of NGOs working on maternal health and family health*, *reproductive health*. *But this is not comparable to the level of problem and demand*, *they are few*, *the problem of the society is large and severe*. *Even the NGOs’ budget amount is not comparable*, *there is a gap between the level of the problem and the budget*. *We need more support internally and internationally*. *Our people especially in the two Borean administrative zones*, *Guji*, *East Bale and the two Hararge are in a difficult situation (draught)*, *even to access food and basic service*, *the current drought is exacerbating this*. *They are in a very difficult situation; their cattle have died*.*” (KII 5*, *Regional health bureau*, *Primary health expert)*.

### Fragmented medicine and medical equipment and supplies

Essential supplies like vaccines, delivery beds, gloves, masks, and other products are essential to provide quality service and enhance MNCH outcomes. CHWs in rural Ethiopia indicated that they are providing vaccines for children under five and Integrated Community Case Management to reduce child deaths from malaria, pneumonia, and diarrhea. However, participants said that most health posts have no vaccine storage/refrigerator as they do not have appropriate rooms and electricity. A district village CHW shared their perspective:

“*The major issues that I can raise as problems are*: *We did not have electricity for vaccination services for women and children*. *We have no refrigerators*. *So*, *mothers and children have to wait for a week until we go to the health center and bring the vaccines for weekly supply*. *Some mothers come from long distances*, *maybe they use public transport*. *If they are not getting the vaccine the first day they have to come back*, *which would incur more expenses*. *Pregnant women cannot be vaccinated unless they visit health centers by going far*. *Also*, *there is a time when Iron supply is short*. *Pregnant women need timely TT* (Tetanus Toxoid) *and Iron but the supply is not consistent*. *“FGD participant*, *Ejere District)*

Participants emphasised fragmentation challenges as shortages of the required medicine and interruption of medicine supply for under five children due to the absence of basic utilities like refrigerators and electricity.

“*We have no refrigerators and electricity to store vaccines*. *There is an interruption of supply*, *sometimes*, *we experience a shortage on the required medicine as we are working on treating children five and under*. *For example*, *in the case of supplies for treating children five and under*, *you may not always have zinc and ORS at the same time*, *the supply is inconsistent*.*” (FGD participant*, *From Adea Berga District)*

CHWs are typically supplied with these equipment and drugs by a primary health center to which they are linked, with this came several major risk factors or underlying challenges, for example shortage in inventory and poor transportation have come into focus for community health program logistics and supply chains.

### Fragmented infrastructures of working and living environment

Infrastructure development can determine the access, coverage, and utilization of basic social services including community health programs. In rural Ethiopia, despite the improvement in infrastructural development over time, disadvantaged rural majority population are not effectively accessing basic primary health due to fragmented development of basic infrastructures like road, electricity, clean water supply, and communications technology. In those areas, access to MNCH services is still limited. Even where services are available, they most likely do not have adequate basic infrastructural prerequisites to function at the very basic level to provide essential services including MNCH services.

Our finding reveals the challenge of infrastructural development fragmentation for effective essential health services. Level four (diploma level) HEWs are supposed to be capable of providing skilled delivery services, but they do not have the appropriate working place and equipment. There were similar perspectives from participants at different levels. Perspectives from regional experts provide further insight:

“*To sum up*, *majority of the health posts are old or decaying*. *In conflict/ war areas*, *they are destroyed*. *Previously constructed health posts are not appropriate to provide the current service demand and a standardized service*. *They do not have appropriate and adequate room for storage*, *treatment*, *environmental sanitation*, *ventilation*, *electricity*, *water*, *or fridges to store vaccines*. *Renovation of the health posts*, *equipping the health posts with standard equipment*, *improved infrastructure*, *water*, *and electricity is very crucial*. *Some health extension workers are leaving their jobs which should be addressed*.*”* ***(****KII 5*, *Regional health bureau*, *Primary health expert)*

“*Those HEWs that have reached level four can perform delivery and other curative services*. *However*, *there are no proper logistics to provide the service*. *There are no proper rooms and equipment to provide delivery services*. *To perform a delivery requires comfortable rooms and a supply of medicine*. *Maybe*, *it is being considered for the future because they need more supply*. *For example*, *they need water*, *electricity*, *roads and so on*.*” (KII 9*, *Zonal level health MNCH expert)*

Health posts are inadequate to provide optimum services, the working situation is not favourable. CHWs are practicing their work in precarious conditions including having to navigate difficult terrain and walking long distances to reach the scattered community residence/area.

“*There are HEWs without residential homes*, *some of the HPs (health posts) are decaying*, *some of them have construction quality issues from the beginning due to poor materials (mud and wood)*. *There is progress in improving the health posts*, *but not enough yet*. *The health posts are not adequate to address the current service demands*, *there are inadequate materials and equipment to provide service*, *all the fridges are not functioning or are inadequate*. *HEWs are expected to go to the nearby health center to store or pick up vaccines*, *this is also impacting the potency of the vaccines*. *The constant move from health posts to health centers*, *health posts to homes is challenging*.” *(KII 5*, *Regional health bureau*, *Primary health expert)*

“*Some of us got the level four college diploma*. *We are trained to provide delivery services*. *Now*, *we are supposed to provide delivery service*, *but in practice*, *we are not doing that*. *There is inadequate supply to manage delivery at health posts*. *We don’t have enough delivery equipment*, *there is no water*, *there is no electricity to undertake delivery*. *Sometimes there is an urgent case and shortage of ambulance service*. *Currently*, *they[women] give birth at the hands of HEWs at health posts*. *Some women come to the health posts at the point of delivery*, *if you refer them to the health posts*, *they might give birth while traveling*, *but this has a risk*. *So*, *to properly serve them there should be enough supply of the required equipment available at the health posts*. *(FGD*, *Adea Berga District*.*” FGD*, *Adea Berga District)*

### Fragmented HEWs/CHWs empowerment

CHWs are amongst the frontline drivers of linking communities, especially disadvantaged communities, with the health system to access essential primary health and MNCH, thereby improving health outcomes. Our analysis indicates that CHWs are experiencing multiple challenges related to fragmentation in the quantity and quality of knowledge, skill, incentive, benefits/ motivations, and job satisfaction. Furthermore, opportunities to upgrade their skills and on-the-job refresher trainings are limited. CHWs have mixed levels of college training, some are on level four and others are still on level three college training.

“*It is impossible to say that their knowledge is sufficient*. *When the program was started*, *they didn’t have enough training*, *it was said ‘Better than nothing’*. *This was to prevent the spread of diseases*. *It was said to be sufficient for teaching or promotional work*. *However*, *now they are also doing treatment work with limited capacity*.*” (KII 9*, *Zonal level health MNCH expert)*

However,

“*The training is not enough*. *All HEWs are not equal*. *Some HEWs are still on level 3*. *The upgrading approach is not fast and transparent*. *They abuse us based on our limited work experience*, *when you ask for trainings or upgrading opportunities*, *they require long work experience*. *No upgrading means no salary improvement or promotion*.*” (FGD participant*, *Ejere District)*

“*The on-the-job training opportunities are not consistent*. *For example*, *while there are two HEWs at the HP*, *training opportunity is given to only one HEW*. *Then*, *this would create disagreements between the person who got the training during practice and the person who did not*. *When the person who got the training is absent the service would not be accessed*. *Such an approach would negatively impact the job*. *So*, *it is better if both can be trained equally*.*” FGD participant*, *Adea Berga District*

Also, CHWs experience restrictions for upgrading or diversifying their training and career progression and long years of service at their current sites. They also experience a lack of important benefits, incentives and motivation. Furthermore, a fragmented empowerment approach and lack of conducive working and living conditions are negatively impacting their job satisfaction and performance.

### Fragmented monitoring, supervision, and information system

Supportive supervision is a vital program management mechanism through transparent communication and team-building approaches while monitoring the quality and quantity of the service to promote compliance with standards of practice. CHWs acknowledge the essentiality of supervision and monitoring to enhance their communication with the health system, and update and upgrade their knowledge, skill, productivity, and performance. However, participants highlighted the potential fragmentation challenges in monitoring and supervision in the CHWs program and primary health program. The major challenge includes a lack of appropriate planning, budget, and transportation/ infrastructures. The current security and politically unstable situation and COVID-19 are worsening these challenges.

There are systemic fragmentation challenges in monitoring and supervision in the CHWs program.

“*I think there is a systemic gap regarding supervision and supportive mechanism*, *follow up is low from the regional health bureau to zonal*, *to the district to our level*. *Every time there is an initiative to start*, *but it is not sustained*. *When the region makes supervision an issue the lower-level health offices make it serious*. *When it is ignored from the top*, *it also ignored at the lower level*. *Regular cooperative planning and implementation of supervision can led to better achievement*.*”* ***(****KII 1*, *MoH*, *Primary health program expert)*.

“*Supervision is important for our work*. *But it is not regular*, *it is off and on*! *From my experience there is no proper supervision*. *Sometimes they come from the health office for other work*, *not specifically to review our work in detail and to give us constructive feedback*. *Sometimes*, *they come to coordinate campaigns*.*” (FGD participant*, *Ejere District)*

Participants indicated that supportive supervision is not yet fully programmed in all levels of the CHWs primary health system. There is no proper plan, it is just performed for formality as an administrative task to audit performance. Currently, there is no specific person responsible for the CHWs program supervision at the district level. It is performed by the higher officer or hierarchy order or pushed to the lower structure.

“*Supervisors expect force*, *push factors from top officials*. *Supervision schedules are only frequent when there is a detection from above the higher-level officials*. *The supervision schedule is not equal for all packages*. *The supervision approach is not based on regular schedule*,*” (FGD participant*, *Ejere District)*

“*In my experience*, *the supervision is a kind of feeling checklist*, *it is not to support us*. *Even the existing supervision is only scheduled and conducted during conducive weather*, *they do not come during rainy seasons*, *it doesn’t include winter season (between June to September)*.*” (FGD participant*, *Ejere District)*

The volatile security and political issues are exacerbating the challenge of inadequate supervision. Some Health facilities have incomplete or some inadequate supervision. Participants indicated a lack of budget for the fragmented monitoring and supervision. Participants opined that the issue of supportive supervision should be emphasized and maintained to enhance better performance and health outcomes.

### Fragmented coordination and governance and security

Health system governance requires health sector and non-health sectors decisive support to ensure the health and safety, and productivity of the people. Systematic health management and leadership are important to ensure appropriate policy framework, allocation of all forms of resource and collaboration at all levels. Proper health management and leadership will also ensure safe working environments, improve productivity and impact using appropriate planning, monitoring, and evaluation frameworks. Participants highlighted multiple challenges of fragmentation while applying the policy to practice.

Participants highlighted the fragmented health system-wide governance and coordination challenges. The existing approach is not effective and there is a challenge to ensure uniform health leadership throughout the health system.

“*This is what makes our country so unique*. *I have seen the experiences of other countries and I say ours is good*. *But I can’t say out loud that the coordination platform is uniform and functional up to the district level*. *It is not possible to say that 100 percent will be implemented as agreed here*, *because the structure below may burden the interests; Or the partners may also make their own interest payments*. *As you said*, *people who are incentivized in connection with poverty and incentives can twist some things*, *so I can’t say that it has been fully implemented*.*” (KII 7*, *MoH*, *Primary health system expert)*

“*I don’t think that there is much of a commitment problem at the higher level*. *The thing is*, *how do we bring it down*? *In what kind of strategy are we going to participate*? *It requires a lot of work*.*” (KII 2*, *National level NGO*, *Program Director)*

The recruitment, assignment, and overburdening of HEWs/CHWs in multiple tasks is a challenge. For example, according to the HEWs policy, CHWs should be recruited from the village they are going to serve and after training they should go back to their village. But, in practice, the recruitment and assignment of CHWs does not always go according to the policy. CHWs/HEWs are also experiencing higher workload and are engaged in multiple health-related and non-health-related sector tasks.

*There is a problem with the recruitment and assignment of CHWs. The strategy says CHWs should come from the village she is going to serve. However, the recruitment and assignment is not according to the guidelines. Also, once they graduate from their training and are assigned to any vacant/open village, they don’t want to stay there. They want to live with their families or in their towns. (KII 4, District health office, HEP experts)*

“*Yes*, *the HEP packages are 18*, *that is many given their number but HEWs are sometimes one or two*. *About 7 out of the 18 focus on maternal and child health*. *Of course*, *overpacking will slow down the performance quality and quantity*. *In addition to those packages*, *they are also working on other government tasks unrelated to health sector activities*. *For example*, *health extensions are part of the local cabinet*. *It also brings problems; it creates an additional workload*. *I think the solution could be increasing the number of HEWs and separating MNCH as a specific program*. *Given the number of activities in MNCH*, *separation of the program is very important*.*” (KII 9*, *Zonal level health MNCH expert)*

Political leaders are inattentive to the working conditions of CHWs and are failing to prioritize the CHWs program. Participants indicated that the prevailing political crisis and instability has been affecting the health sector. Participants also indicated the challenge during planning and performance evaluation.

### Fragmented community and stakeholders’ engagement

Community and stakeholder engagement is about partnership building, it is becoming a buzzword in different aspects of social and environmental development issues. Engagement is increasingly highlighted in Ethiopia’s health policy reforms, HSTP-II [11], HEP optimization [50] and more. Despite the contribution of engagement to the improved health outcomes, the findings indicate that there is a fragmentation and lack of sustainability in the existing engagement approach.

#### Community Engagement (CE)

Participants from different levels shared the same perspective, they uncovered the fragmentation challenge in community engagement. At the federal level, they described how political instability and conflict has set back a lot of things including CE, and with this comes certain gaps that need to be addressed. At the regional, zonal, and district levels, they describe weak community participation due to lack of attention, COVID-19, and volatile security issues.

“*For example*, *the conflict that started two years ago has set back a lot of things*. *It has had a big effect*. *We can clearly see that it has pressure not only on health but also on other sectors*. *However*, *our community engagement work has been good*, *especially since the health extension program was launched*. *Given the current challenge*, *there are certain gaps that need to be addressed*.*” (KII 7*, *MoH*, *Primary health system expert)*

“*Currently*, *community participation is very weak*. *To speak honestly*, *unstable security is an issue*, *it is a big challenge now*. *The attention of the community is on security issues*, *not on the health or other public service*.*” (KII 5*, *Regional health bureau*, *Primary health expert)*

Currently, the community structure is not functioning as it was intended. It is characterized by considerable dropouts or interruptions. Its strategic direction is not well organized, planned, and regular.

“*Nowadays*, *community structure is not functioning well as it was intended*. *It is characterized by considerable dropouts or interruptions*. *Now*, *it is interrupted and there is a dropout as the people developed negative attitudes towards such structure as some times the way of organization and participation is attached to political activities and orientation*.*” (FGD participant*, *Adea Berga District)*

“*This time the focus of the leadership is on the current political conflict issue and there is no organized approach from the community*. *In some areas communities have great motivation*, *they are contributing resources to build health centers*. *In some districts*, *the community bought 2–3 ambulances*. *There is a big motivation in some areas to meet their service demand*. *(KII 5*, *Regional health bureau*, *Primary health expert)*

“*The community raises many concerns about health center and hospitals*, *they say there is not enough service there*. *When we mobilize the community to organize crops for delivery and Community-based health insurance*, *they are not willing to actively participate*, *because they have a concern about the service*, *they are getting from health centers and above*. *As a solution*, *quality service should be ensured for all community members to enhance community participation in health programs*.*” (FGD participant*, *Adea Berga District)*

#### Stakeholders’ engagement

In Ethiopia, despite the commitment to strengthen multi-stakeholder engagement and optimize the community health program in the national level health policy, our findings reveal multiple challenges at the implementation level. For example, there is a lack of system strengthening or holistic support from stakeholders or actors. Engagement of the private sector is minimum in the CHWs program. These have resulted in gaps between policy and practice.

“*There are limited NGOs/CSOs in our area compared to the need*. *Due to security challenges*, *some of them are leaving their site including public health facility workers*. *Some partners have resource constraints*. *Some of them shifted their resources to COVID-19 response*.*” (KII 10*, *Regional NGO*, *Program manager*)

“*A partner who comes to support the whole health extension program is very limited*. *Partners are interested in specific intervention to provide onsite or offsite trainings*. *There is a mismatch in actors’ programs*. *For example*, *HEP requires holistic intervention*. *However*, *NGO actors come with specific programs*. *They could come just for EPI; they have the challenges to support the whole program*. *When a partner comes without system supporting capacity*, *it doesn’t strengthen the program*. *The government does not want this*, *and that is one of the challenges*. *And one of the discussions was that things like this should be fixed*.*” (KII 1*, *MoH*, *Primary health program expert)*

In the macro-level policy documents, like HSTP-II [11], a roadmap for optimizing the Ethiopian HEP(2020–2035) [50] and more (see S2 File) health finance, medical and equipment supply, health infrastructures, HEWs/CHWs empowerment, supervision, information system, governance, and community and stakeholder engagement are among the major issues of the health sector. However, participants emphasised that despite having these themes as major issues to be addressed by the government in policy documents, they were not changed in practice.

## Discussion

In LMICs including countries like Ethiopia, community-based health programs are a cost-effective way to extend health services to hardest-to-reach communities and improve their health outcomes. They are also essential to accelerate progress toward national and global health goals including MNCH. In Ethiopia, the CHWs program is a part of the primary health care system and has been instrumental to the disadvantaged rural communities with essential primary health services and in the recent improvement of health outcomes including MNCH. In the country, despite significant progress in expanding community health services, gaps remain in improving access to MNCH for populations. We conducted a qualitative case study in Ethiopia to analyze comprehensive evidence on the paths to transform the CHWs program to enhance MNCH equity and a resilient community health system in rural Ethiopia.

Using thematic analysis major themes on challenges of fragmentation of community health program pillars emerged. We identified seven distinct yet interconnected sub-themes (sets) of fragmentation challenges in the community health program pillars and systems. These multidimensional challenges of fragmentation include fragmented health finance, fragmented medical and equipment supply, fragmented working and living infrastructures, fragmented CHWs empowerment and motivation, fragmented monitoring, supervision and information, fragmented coordination and governance, and fragmented community and stakeholder engagement. Our finding underscored fragmentation as a blind spot for most of the ambitious primary health policies and strategies of Ethiopia. The study emphasised the gap between the policy at the macro (national) level and the challenge during implementation at the micro (district) level. The ongoing COVID-19 pandemic and volatile political and security challenges in the country are exposing and exacerbating the fragmentation challenges.

The result suggests that all the multilevel participants perceived multidimensional challenges of fragmentation in the CHWs program to deliver equitable, sustainable, and quality MNCH in rural Ethiopia. Here, it is necessary to interrogate what we mean by “fragmentation” in the CHWs program. Health system can be compromised in fragmented systems, health service users experience lack of access to optimum and comprehensive services, loss of continuity of care, and the failure of health services to meet their satisfaction [26]. Fragmentation may undermine health system quality and efficiency. Fragmentation could occur due to fragmented actors, inadequate governance arrangements, inadequate financial planning, misalignment of empowerment, and duplication and mistargeting of services [59]. It can create health system gaps and adversely affect economically disadvantaged communities [29].

The CHWs program supply-side fragmentation challenges have affected and continue to affect the MNCH and primary health access in rural Ethiopia and beyond. Our analysis reveals that the CHW program is inadequately funded. Inadequate health financing is the most common challenge that is keeping national CHW programmes from reaching their full potential. Although fragmentation of health financing is well documented in the literature [21, 30, 33, 60], financing of national health programs has been a critical element that has not received sufficient emphasis among decision- and policy-makers.

In 2020, the WHO reported that the critical health system challenge for this decade is that global leaders are failing to invest enough resources in core health priorities and systems [61]. A recent study in Zambia and Ethiopia [62] shows that inadequate health financing is a critical challenge to strengthen CHWs program and ensure MNCH and a resilient community based health system. Sixty percent of funding for CHW programs in SSA is from donors, and most of this is for vertical disease-specific programmes [63]. In Ethiopia, there are inconsistences in government spending in primary health. Similarly, in the country, the proportion of HEP expenditure out of the total expenditure and total primary health level spending has dropped [50]. In SSA national spending on health is still low, only four of 55 countries in the African Union have reached the 15% commitment, which was set in the Abuja Declaration in 2001 [64]. LMICs aiming to build health systems that are publicly funded are largely influenced by external aid objectives and under the power of donor-driven funding delivered in a fragmented way [63–65].

Genuine and practical political commitment is a key prerequisite to addressing the health financial challenge and scaling up the available resource mobilization options. Leveraging evidence-based, systemic, and practical domestic resource mobilization including scaling up the different insurance scheme initiatives aligned with the diverse living and working context of the general population and rural community. It is also important to organize evidence on the corporate social responsibility model [66] for the private sector to take responsibility in community health while refocusing funding from the external resource on whole health system strengthening. Fragmentation of health financing is the existence of a large number of separate funding mechanisms (e.g. many small insurance schemes) and a wide range of health-care providers paid from different funding pools [67].

Lack of necessary supplies can compromise health quality and cause disruption. Participants in the study also reported that fragmented medicinal and equipment supply are serious challenges to delivering optimum MNCH. FGD participants, CHWs, revealed that most health posts have no vaccine storage/refrigerator as they do not have appropriate rooms, or basic utilities like refrigerators and electricity. Evidence from different countries including Ethiopia and Sierra Leone shows that the crumbling state of supply of such essential medicines and equipment is a major impediment to their effectiveness and motivation [13, 25, 50, 68]. Lack of investment in medical and diagnostic supplies has made it harder for African nations to effectively respond to MNCH and other essential health issues [15, 16, 25, 69].

In rural Ethiopia, despite the improvement in infrastructural development over time, disadvantaged rural majority population are not effectively accessing basic primary health due to fragmented development of basic infrastructures like road, electricity, clean water supply, and communications technology. In those areas access to MNCH services is still limited. In SSA, one of the biggest healthcare challenges rural residents face is the lack of reliable transportation and ambulance service, a low level of infrastructural support, and the absence of adequate health facilities in their neighbourhoods [16, 68]. In SSA the poor level of health infrastructure in countries such as Tanzania, Zimbabwe, Kenya, and Nigeria has been linked to a lack of long-term investment [15, 16, 69, 70].

Multilevel barriers to CHWs’ effectiveness in SSA include fragmentation of empowerment of CHWs programmes [16, 69]. In Ethiopia, there is a shortage of the required capacity of CHWs to effectively deliver expanded HEP packages [50]. Recent studies in Bangladesh, India, Kenya, Malawi, and Nigeria report similar findings and indicate that inadequate knowledge affected service delivery and raised questions about the quality of CHW services. CHWs’ insufficient knowledge was partly explained by inadequate training opportunities and the inability to apply new knowledge due to equipment unavailability [68]. A study in Cambodia [65] and Iran reveals CHWs experience high workloads and lack a support system. These were mentioned as barriers to their effective performance [71]. There is a need to review and revise their scope of practice to reflect the varied duration of training [72]. CHWs require the formulation and implementation of policies that support their work, as well as financial and nonfinancial incentives, motivation, collaborative and supportive supervision, and a manageable workload [73].

Insufficient and lack of continuity of coordination and governance are common challenges in SSA CHWs/MNCH programme [16]. There are multiple actors engaged in supporting CHWs program without national or local mechanisms for coordination [74]. For example, actors/donors promote vertical programs and disease-specific programs [27, 74]. Multiple actors are acting on the parts without adequately appreciating their relation to the evolving whole [31]. Schneider and Nxumalo (2017) [75] explore the leadership and governance tasks of large-scale CHW programmes at the sub-national level in South Africa’s CHWs strategy. In their finding, to overcome leadership and governance in CHWs program: alignment of national mandates to provincial and to district strategies; integrating planning, human resource, financing, and information systems; redesigning organisational and accountability relationships between CHWs, local health services, communities and CSOs [75].

Participants opined that the issue of supportive supervision and data systems should be emphasized and maintained to enhance performance and health outcomes. A recent systematic review and synthesis of the literature on HEP in Ethiopia between 2003 and 2018 [13] has indicated the lack of adequate supportive supervision [13]. Fragmented supervisory support could be associated with a lack of motivation and capacity gaps in supervision [76, 77]. Fragmented supervision of CHWs has been a challenge for large-scale national programmes due to the lack of systemic supportive supervision approaches [78]. A review synthesising the evidence of CHWs’ experiences of supervision in LMIC emphasized best practices of effective supervision time, resources, and supervisors’ skills and knowledge. Employing supervisors whose sole responsibility is to supervise CHWs may be a good strategy to the alleviate aforementioned issues faced by CHWs [79].

Stakeholders’ engagement is increasingly highlighted in Ethiopia’s health policy reforms [11, 50]. Participants reported that there are multiple platforms of community and stakeholder engagement in community health, however there is a lack of consistent engagement during planning, implementation, and evaluation across the different packages of community health and different health administration structures. In addition, we argue that despite the multiple fragmented platforms of engagement [50], there is inadequate emphasis on meaningful engagement towards a coproduction framework including co-planning, co-implementing, and co-evaluation of health programs at all levels.

### Policy implications

Fragmentation by itself or in conjunction with other factors can lead to complications in accessing optimum and comprehensive services [26]. Fragmentation may undermine health system quality and efficiency, exacerbate health extreme inequalities, and inequitable access to healthcare. In Ethiopia, it is continuing as a threat to CHWs program gains, sustainability, and resilience of the MNCH and primary health sector in rural Ethiopia in part due to fragmentation and absence of adequate fragmentation monitoring and maintenance.

Currently, optimization of HEP is acknowledged and increasingly recognized as an important issue in the primary health policy agenda through task shifting and professional mix to achieve the SDG and UHC #3 [50], however, the fragmented situation has not changed yet. Our finding shows that there is a high risk that if the situation continues in a business-as-usual mood, community health program pillars fragmentation will continue to impede community-based MNCH outcomes and health system resilience efforts.

Remedial solutions for overcoming fragmentation blind spots in CHWs program should consider the following: An in-depth understanding of the problem is crucial to acknowledge the problematic nature of fragmentation in primary health policy and practice. Evidence-based and systemic fragmentation monitoring and responsive approach is crucial to achieve better health outcomes and ensure a resilient community health system to improve MNCH, to achieve UHC and the SDG #3. Finally, there is an urgent need for transformative and innovative approaches that capitalize on stakeholders’ engagement in the context of coproduction of improved MNCH outcomes and a resilient community-based health system through transformative system thinking. These are especially pertinent in light of the challenges faced and lessons learned in the context of the COVID-19 pandemic, which showed that fragmentation is one of the main institutional barriers to addressing the population’s health problems [27].

### Strengths and limitations

To the best of our knowledge, no study has examined the multifaceted challenges in CHWs/MNCH program in Ethiopia using multilevel and multidimensional data. This is the first qualitative case study using multilevel perspectives of national, regional zonal, and district policy actors, and onsite CHWs. The research team has extensive experience as global health experts and have multiple publications and work on MNCH and related policy and programs in SSA including Ethiopia.

This study has two potential limitations. First, this is a case study, which will not be generalized for all the CHW programs in urban and rural areas of Ethiopia. Second, the community health program in Ethiopia is predominately designed to be run by females only, hence this study did not address men’s’ and urban HEWs perspectives. Due to prevailing security issues and political mistrust within the community, KII participants from zonal and district levels and some CHWs appeared to show some level of frustration and caution in fully expressing their opinions, therefore key perspectives might have been omitted from their responses.

## Conclusion

In Ethiopia, although nationwide community-based health program is available in every rural village, the health program has been facing unique system challenges and fragmentation. The study emphasised the systemic gap between the policy at the macro (national) level and the challenge during implementation at the micro (district) level. Fragmentation is a blind spot for most of the ambitious primary health policy strategies of Ethiopia. This is a threat to sustain the MNCH outcome gains and resilience of primary health access in rural Ethiopia. We argue that the challenges of the program fragmentation are exacerbating the fragility of the health system and fragmentation of MNCH health outcomes like ANC, SBA and PNC. This can aggravate extreme health inequalities and health inequities between rural (areas covered by CHWs) and urban. Rural communities especially mothers and children, on average, would get less access to optimum and quality healthcare services than their urban counterparts.

We suggest that beyond efforts to expand the service coverage, the government (health system) and the public health partners (actors) need to problematize and understand the challenges of fragmentation in the community health system and respond to challenges through enhanced multi-stakeholder engagement in the context of a coproduction framework. Additionally, a socio-ecological framework and an intersectionality lens should be given priority to guide the understanding of the causes of fragmentation challenges and the complex ways in which multilevel barriers to equitable, resilient, and universal CHWs/MNCH service relates, intersects, and mutually reinforces one another.

National health program fragmentation is less acknowledged among researchers and policy actors while often from a high-level perspective, it has been discussed in the global health literature, agendas and between prominent global health actors.

## Supporting information

S1 File Consolidated Criteria for Reporting Qualitative Research (COREQ). (DOCX)

S2 File List of Ethiopian HEP/MNCH related policy documents. (DOCX)

S3 File Additional quotes. (DOCX)

## Abbreviations

CE: Community Engagement
CHW: Community Health Work
CHWs: Community health workers
CSO: Civil -Society Organization
FGD: Focus Group Discussion
FMOH/MoH: Federal Ministry of Health
HEWs: Health Extension Workers
HSP: Health Sector Development Program
HP: Health Post
HSTP: Health Sector Transformation Plan
KII: Key informant In-depth Interview
LMICs: Low- and Middle-Income Countries
MDG: Millennium Development Goal
MDGs: Millennium development goals
MMR: Maternal Mortality Rate
MNCH: Maternal Newborn and Child Health
NGOs: Non-Governmental Organization
SDGs: Sustainable Development Goals
U5MR: Under Five Mortality Rate
UN: United Nations
WHO: World Health Organization
