# Supplementary material for: Unpacking the challenges of fragmentation in community-based maternal newborn and child health and health system in rural Ethiopia: A qualitative study
Source: PLoS One. 2023 Sep 21;18(9):e0291696. doi: 10.1371/journal.pone.0291696 (PMC10513239; doi:10.1371/journal.pone.0291696)
Supplement: S3 File — (DOCX) [file pone.0291696.s003.docx]

**S3.** Additional quotes

We present the seven distinct yet interconnected sub-themes (sets) of fragmentation challenges in community health program pillars.

| **Sub-themes** | **Description and quotes** |
| --- | --- |
| 1. **Fragmented health finance** | Participants explained that the Ethiopian health system is dependent on donor support. Despite the higher demand of funding due to the prevailing problem, including high maternal and child morbidity and mortality, the available funding is fragmented, small, declining, and project specific. Also, there is a lack of transparency and duplication in the management of available funds.  *“There is a general understanding that the health sector has more budget which is from donors. …But according to the recent National Health account assessment, the government budget allocation is increasing, and donor support is declining. Compared to the Abuja declaration of 15% budget allocation out of the GDP, ours is still below 10%. This has an impact on the health service and infrastructural development. Sometimes, I review the budget allocation, so that the highest share of the budget is allocated to MNCH. Donors also have a high interest in this area. But still, the budget gap is becoming an issue and there is a plan to present to the parliament a request to improve the budget allocation. The budget gap is everywhere but may not be significant as in health. The budget gap can affect the service, so it is important to prioritize the service provision until a sufficient budget is ensured. MNCH cannot be compromised because it is a sensitive issue. It is not like a communicable disease.”* (KII 7, MoH, Primary health system expert).  Budget allocation was said to vary based on the different levels of leadership commitment and understanding of CHWs / MNCH challenges.  *“In my current evaluation, the government has its own priority. Even the leadership approach is like a campaign. Sometimes they give more attention for example for MNCH, they initiated some moto for this part of health activities “mother should die while giving life.”. But it has no continuation, it will decline or be politicized. There is no regular support. Even if you take and see the district level, these differences exist. When you see the district-level health sector some districts have better government budget allocation and others do not have enough budget from the government treasury. Then, when you ask them why? Because health has NGOs, NGOs can finance health. This is a management problem in some areas. When the leadership speaks for health, there is better allocation of resources for health.” (KII 10, Regional NGO, Program manager).*    Furthermore, participants contended that a lack of reliable budget is impacting the service coverage and quality. It requires improving expenditure to the primary and community health program.  *“Yes exactly, there is not enough funding or budget allocated for CHW program. There is limited support, so you will be restricted to provide optimum service.” (KII 3, district level CHWs program expert).*  Budget allocation was said to vary based on the different levels of leadership commitment and understanding of CHWs / MNCH challenges.  *The government’s commitment is not consistent. …Political leaders expect the health officials to take all the responsibility, they assume the health office have better sources of funding.” (KII 3, District health office, HEP experts).* |
| 1. **Fragmented medicine and medical equipment and supplies** | CHWs indicated that stock-out of such essential medicines and equipment is a major impediment to their effectiveness and motivation.  *“We are working on Implant and Intrauterine devices (IUDs), the supply is not consistent. There is also no regular supply for under-five children treatment.”* (FGD, Adea Berga District).  *“Besides Covid-19, we have challenges related to supply. MNCH is free in our area, but due to supply issues sometimes there is a possibility of referral to private hospitals. The other challenge is lack of Ambulance and lack of ambulance maintenance.”* (KII 3, District health office, HEP experts). |
| 1. **Fragmented infrastructures of working and living environment** | Even where services are available, they most likely do not have adequate basic infrastructures prerequisites to function at the very basic level to provide essential services including MNCH services. CHWs are practicing their work in precarious conditions including having to navigate difficult terrain and walking long distances to reach the scattered community residence/area. The lack of efficient modes of transpiration and poor infrastructure for personal mobility and transportation of work equipment is impacting job satisfaction.  *“However, there is a lack of infrastructure, buildings of health organizations; There is a lack of budget to hire a health professional. Currently there is a security problem to implement the service.”* (KII 9, Zonal level health MNCH expert, KII 4, District health office, HEP experts).  “*They don’t have satisfaction with their job, some of them are also leaving their job and trying to find jobs in other sectors. The infrastructure is gradually improving of the curse, but it is not sufficient for their work. Most of them don’t have logistics, no transport system, no water, no electricity.”* (KII 10, Regional NGO, Program manager).  *“In some zones, they can even travel an hour during outreach work for this they don’t have motorbike or transportation facility.”* (KII 5, Regional health bureau, Primary health expert).  HEWs have no conducive living or restroom around their work site. They have no guarantee for their safety and security. They live and work under poor living conditions.  *“CHWs have multiple living challenges. For example, in terms of housing (place of residence). We work in a difficult and remote geographic area (there are ups and downs to finding and addressing the scattered rural villagers), our work is mostly outreach and home-to-home, and we have no transport access. We just do our work by walking. We have a security issue in the district, and as a woman, we have fears too. We have no facilitators to mobilize the community during different outreach activities. We have a lack of transportation when we return from the Woreda/ district after training and other meetings.”* (FGD, Adea Berga District).  *“I can mention some of the major health system challenges. First there is low infrastructure. The ratio of population to infrastructure are not comparable, it’s very low. Second the health institutions are very old and are decaying, there is a design problem to provide appropriate service. The third one is the lack of the required equipment to provide quality health care service, for example there is inadequate BP apparatus. There is a challenge to purchase and fill the supply gap due to financial challenge.”* (KII 9, Zonal level health MNCH expert).  *“HEWs have no living or restroom around their work site. The health post is not sufficient and conducive for our work. As any human being, we deserve to rest after precarious work. Some of us who are living in small towns should travel between our home to the work site, and some of us are living in a rental home. I used to live in a house built for a teacher and agriculture extension worker, even these houses were not adequate for living.”* (FGD participant, Ejere District).  *“There is a place where there is no health post and some health posts are decaying, aging. So, it is important to improve the health post status. Supply is also another challenge to providing proper service, so availing adequate equipment is important.”* (KII 9, Zonal level health MNCH expert). |
| 1. **Fragmented HEWs/CHWs empowerment.** | CHWs are experiencing multiple challenges related to fragmentation in the quantity and quality of knowledge, skill, incentive, benefits/ motivations, and job satisfaction while exciting these essential services CHWs.  *“For example, there is a big gap between the work we do and the salary we get. We undertake more than 16 packages. The package and the health extension workers’ roles are increasing every time. We do family planning activities. So far ANC service was up to 4, but now it is up to 8 rounds. The training and the job load and the benefit are not comparable. Even the training given is not enough. The reporting system is changing but we don’t have enough training and skills.”* (FGD participant, Adea Berga District).  *“The benefit and the job burden for the HEWs are not comparable, the distance like from ‘the ground to the sky’. Our benefit is very low, we serve many communities, it took us a long time to convince a single mother. Our salary and workload are not comparable. It is not adequate for living and supporting a family.”* (FGD participant, Ejere District).  *“The health system officials need to consider the condition of HEWs, other health workers have the option to study in different way, privately or by government, even outside of the health program. However, HEWs have no options, they are not allowed to study outside of the HEP or the cant study privately, it is not accepted in this sector. Health extension career path is not flexible, there is no option to request promotion to relatively better work site, beyond their village. It’s boring for HEWs to stay at the same site for their entire job career life, we need change.” (*FGD participant, Ejere District). |
| 1. **Fragmented monitoring, supervision, and information system** | Participants highlighted the potential fragmentation challenges in monitoring and supervision in CHWs program and primary health programs. The major barrier includes the lack of an appropriate plan, budget, and transportation/infrastructure. The current security / political stability and COVID-19 are worsening the challenges.  *“Another challenge is in relation to the health extension and health care linkages. There is a structure, to support the CHWs. For example, HC staff is expected to provide support to the health post, but there is no regular support, or the staff does not have the required preparation and capacity.”* (KII 10, Regional NGO, Program manager).  *“So far, the support system from the upper health system is inconsistent, it should be emphasized and maintained. Without strong respective support, our performance would be in question. It is important to strengthen smooth and sustainable relationships. Regular or monthly and friendly monitoring and supportive mechanism are crucial. If so, no way of lagging. Supervision is very important to refresh, update, give focus to, to be on track.”* (FGD participant, Adea Berga District).  Participants indicated that supportive supervision is not yet fully programmed in all levels of the CHWs/ primary health system. There is no proper plan, it is just performed for formality as an administrative task to audit performance. It is performed by the higher officer or hierarchy order or pushed to the lower structure.  *“Most supervisors come without preparation, prior notice and they give verbal feedback, they do not give written feedback which could be a good document to refer to.”* (FGD participant, Ejere District).  *“Most of the time they do not prepare themselves for the task, when they arrive here, they do not provide feedback. Especially for those who come from the zone, their feedback is limited to oral than in writing. It is good if they provide written feedback on strong and weak performance. Also, we can prepare ourselves if they orally receive our opinion with an appropriate response. Hence, we will be ready and proceed accordingly.”* (FGD participant, Adea Berga District).  The volatile security and political issues are exacerbating the challenge of inadequate supervision.  *“On top of COVID, due to the current security issues, we are only supporting two health centers (Inchini and Reji), we go to “Reji’ health canter, but we cannot go to all the respective health post areas. We are afraid that we are not going to Kerkeresa and Olonkomi area health centers and their respective health post, the service is mostly interrupted. All others are beyond our control.”* **(**KII 3, District health office, HEP expert).  *“The problem in our zone due to the current security issue is supportive supervision and supply distribution are interrupted. Most health extensions are not on site due to security; during the winter season it is difficult to provide supportive supervision due to the lack of transportation; Currently, the supportive supervision is only for the site on the main road.”* (KII 9, Zonal level health MNCH expert).  Participants indicated a lack of budget for the fragmented monitoring and supervision. Currently, there is no specifically responsible person for CHWs program supervision at the district level. Participants opined that the issue of supportive supervision should be emphasized and maintained to enhance better performance and health outcomes.  *“CHWs/ HEWs program monitoring and supervision is left behind due to budget shortage. Experts are not going down to support the health extenuation program. There is pressure from higher officers toward the district. But to conduct consistent support, many things could be fulfilled to keep them going. For example, when you get funds, you can go to the bottom/ lower level HEWs site for support. You can be aware and support the staff.”* (KII 3, District health office, HEP expert).  There is a security challenge.  *“To be frank in last three years in west Ormiya, it is difficult to travel from one site to another site a big supply challenge. because of the security issue. Even we are not distributing supplies, mobility is restricted in those area. Because of this, for example if I can call to one district just know they are in This are our challenge. This has affected our work, HEWs work and the public in general.”* (KII 10, Regional NGO, Program manager).  *Budget shortage.*  *“CHWs/ HEWs program monitoring and supervision is left behind due to budget shortage. Experts are not going down to support the health extenuation program. There is pressure from higher officers toward the district. But to conduct consistent support, many things could be fulfilled to keep them going. For example, when you get funds, you can go to the bottom/ lower level HEWs site for support. You can be aware and support the staff.”* (KII 3, District health office, HEP expert). |
| 1. **Fragmented coordination and governance and security** | Participants highlighted the fragmented health system-wide governance and coordination challenges. The existing approach is not effective and there is a challenge to ensure uniform health leadership throughout the health system.  *“On the part of the leadership, they believe that improving the health of mothers and children is an essential component for the country’s development. At the system level, there is a plan but the challenge in implementing the plan, the challenge is converting the plan into action. There is a system from MoH to the health center, but in practice, it is not functioning*. *But they* *are not getting optimum support from the government and the partners, maybe this is due to the current unstable security issue. There are policies being enacted to strengthen the health HEP, to reorganize the HEWs program as merged or comprehensive. But it was not practiced, there is no allocated budget.”* (KII 9, Zonal level health MNCH expert).  *“I don't think that there is much of a commitment problem at the leadership level. The thing is, how do we bring it down? In what kind of strategy are we going to participate? It requires a lot of work.”* (KII 2, National level NGO, Program Director).  The recruitment, assignment, and overburdening of HEWs/CHWs in multiple tasks is a challenge. For example, according to the policy CHWs should be recruited from the village they are going to serve, and after training, they should go back to their village. But the recruitment and assignment of CHW are not always according to the plan. CHWs/HEWs are also experiencing higher workloads and have been engaging in multiple health-related and beyond-the-health sector tasks.  *“Health extension workers have multiple barriers to performing their task. HEWs hold a big burden! We got a minimum payment. If you take a health center, every profession works in a different department. But, in our case HEWs work on all packages: child treatment, vaccination, sanitation, delivery, ANC, COVID, and the like. These all jobs are loaded on two or sometimes one HEW, normally in most cases, there is only one health extension worker available at the health post.”* (FGD participant, Adea Berga District).  *“HEWs have been engaging in multiple tasks beyond the health sector task. They were involved in politics, every sector was directing them, and they seem no primary-led sector. They are expected to deliver tasks beyond their role as HEWs.”* (KII 5, Regional health bureau, Primary health expert).  Lack of political leaders’ attention and prioritization to improve the working condition of the CHWs program. Participants indicated that the prevailing political crisis and instability have been affecting the health sector. The health sector is not providing optimum MNCH and other basic services because some health facilities have no staff, no supply, and no supervision. Also, some are looted, burned, or shifted to military camps. Participants decry the lack of political leaders’ attention and prioritization to improve the working condition of the CHWs program.  *“The health sector is particularly affected by the political crisis in the country. If there is no healthy person, he will not participate in agriculture, he will not participate in education, and malnutrition has become worse than before due to the conflict. Another is that even the spread of infectious diseases, which we say we have done well, even if we have not completely won, is currently showing an increasing trend.”* (KII 7, MoH, Primary health system expert).  *“Currently, there is a hidden security issue in the western Ormiya part. Some of them have no interest because they have been there for a long time as there is no transferring mechanism from site to site or district to district. They don’t have satisfaction with their job, and some of them are also leaving their job and trying to find jobs in other sectors. The infrastructure is gradually improving the curse, but it is not sufficient for their work. Most of them don’t have logistics, no transport system, no water, no electricity.”* (KII 10, Regional NGO, Program manager).  *“But they are not getting optimum support from the government and the partners, maybe this is due to the current unstable security issue…Due to security issues about 160 health posts and 10 health centers are out of service due to the security issue/ conflict. Lotted, burned, or shifted military camp, Fidgets are out of service.”* (KII 9, Zonal level health MNCH expert).  Participants also indicated the challenge during planning and performance evaluation. There is more reliance on census-based population projects during annual planning and performance evaluation. There is also a lack bottom to up grass root voice in planning.  *“Sometimes, the plan and the reality do not match. The plan comes from the top, regional bureau. For example, while the actual data I have is 400 children for vaccination, during the performance they do not accept. The same thing to maternal health. There is a weak bottom-to-up approach to planning. Therefore, most of the time, our performance and plan are not matching. There should be a mechanized plan based on the actual data on the ground with the community. Many times, I’m very sad, because they evaluate your performance based on the top to down plan, and the plan doesn’t match the actual. When they evaluate this way, your performance is low always. They don’t accept your justification. They plan based on estimation without the actual data.”* (FGD, Adea Berga and Ejere District).  *“It would be good if the correct information is available from the society. But there is a dependence on Census while planning. The plan is top to down. I believe that the information obtained directly from society should have been used and the plan should have been from the bottom to up. If not, it will not be effective, and the plan may be over or under.”* (KII 9, Zonal level health MNCH expert).  *“It would be good if the correct information is available from the society. But there is a dependence on Census while planning. The plan is top to down. I believe that the information obtained directly from the society should have been used and the plan should have been from the bottom to up. If not, it will not be effective, and the plan may be over or under.”* (KII 9, Zonal level health MNCH expert).  Lack of political leaders’ attention and prioritization to improve the working condition of CHWs program.  *“But it is not being done at the level it should be done. Because the leaders have no attention for the where and how, health extension workers are leaving. This is why HEWs are discouraging and moving to the nearby town / or their family, due to lack of residence or insecurity. There is not enough budget to improve the conditions of HEWs…* *may be this is due to the current unstable security issue.”* (KII 9, Zonal level health MNCH expert).  *“…This time the focus of the leadership is on the current political conflict issue and there is no organized approach from the community.* (KII 5, Regional health bureau, Primary health expert).  Participants also indicated the challenge during planning and performance evaluation. There is more reliance on census-based population project during annual planning and performance evaluation. There is also a lack of bottom to top grass root voice in planning.  *“Sometimes, the plan and the reality do not match. The plan comes from top, regional bureau. For example, while the actual data I have is 400 children for vaccination, during performance they do not accept. The same thing for maternal health. There is a weak bottom to up approach on planning. Therefore, most of the time, our performance and plan are not matching. There should be a mechanize to plan based on the actual data on the ground with the community. Many times, I’m very sad, because they evaluate your performance based on the top to down plan, the plan doesn’t match to the actual. When they evaluate this way, your performance is low always. They don’t accept your justification. They plan based on estimation without the actual data.”* (FGD, Adea Berga and Ejere District). |
| 1. **Fragmented** **community and stakeholders’ engagement** | Despite the contribution of community and stakeholders’ engagement in the improved health outcome, the findings indicate that there is a fragmentation and lack of sustainability in the existing engagement approach.  “*For example, the conflict that started two years ago has set back a lot of things. It has had a big effect. We can clearly see that it has pressure not only on health but also on other sectors. However, our community engagement work has been good, especially since the health extension program was launched. However, given the current challenge, there are certain gaps that need to be addressed.”* (KII 7, MoH, Primary health system expert).  Currently, the community structure is not functioning well as it was intended. It is characterized by considerable dropouts or interruptions. Its strategic direction is not well organized, planned, and regular. It is just a campaign but there is a lack of consensus and lack of trust from the community in the community participation approach. It was not designed based on volunteer bases and currently, the community has no positive attitude. Furthermore, lack of leadership focus and quality service are affecting CE.  *“Community engagement strategy is not well organized. There is some community participation for times when the higher-level leaders give attention or direction it would be hot. It is just like a campaign; sometimes strong time is very low. The overall approach of community engagement is not planned and regular. The community has doubts, and a lack of trust from the community, when you invite the community for different health issues, they assume that there is an attached political issue, so they are not willing to participate when you engage the community on health there is also political issues, so the community doesn’t what this, the community do not engage. There is Community structures are not strong because there is political instability. Community engagement is attributed to some incentives, when there is incentive community engagement is high, and society demands incentives, and refreshments, despite the govt policy of no incentives. The expectation of incentive is a challenge for community engagement, partners have a contribution to this.”* (KII 10, Regional NGO, Program manager).  *“From the beginning, I don’t think the community participation approach was designed on volunteer bases. If this was the case, we wouldn’t have such an interruption. Community participation is still high on other issues like settling disputes related to farmland boundaries and gathering land, however, their participation in health, education, and other government program is not functioning. The approach is not on volunteer bases, there is some political or administrative force behind it. The community has its own organization like ‘idir’or ‘iqub’ they are very committed to those. To improve the community participation.”* (FGD participant, Adea Berga District).  Stakeholders’ engagement: our finding reveals multiple challenges at the implementation level. For example, there is a lack of system strengthening or holistic support from stakeholders or actors.  *“Until last year, World Vision was with us. There were others. For example, there are PSI, PLAN, etc. We have 35 health posts; they have been working on 16 of them. However, almost for more than one year now, we are not supporting them. Although some want to support, due to security concerns they are not supporting. It is a survival issue, your plan to work after your survival. They want to work in a safe area, no one needs to go to an unsafe area.”* (KII 3, District health office, HEP expert).  *“Currently there is no or limited opportunity for the private sector to engage in the community health program. But for the future, there should be a strategy to engage them they have the potential to support in capacity building, logistics, and the like as a social responsibility.”* (KII 9, Zonal level health MNCH expert).  *“But we have not used the private sector very well. Privates also have a social responsibility, don't they? For example, they go into the community and train health extension programs, they help by scaling up their facilities by training professionals. The government is losing very competent professionals, and there are also push factors, so they go from government to private. This means that those who understand the community health program well.”* (KII 7, MoH, Primary health system expert). |
